# Supplementary material for: Rosmarinus officinalis L. Leaf Extracts and Their Metabolites Inhibit the Aryl Hydrocarbon Receptor (AhR) Activation In Vitro and in Human Keratinocytes: Potential Impact on Inflammatory Skin Diseases and Skin Cancer
Source: Molecules. 2022 Apr 13;27(8):2499. doi: 10.3390/molecules27082499 (PMC9029298; doi:10.3390/molecules27082499)
Supplement: Supplementary file 1 [file molecules-27-02499-s001.zip › molecules-1638006-supplementary.pdf]

SUPPLEMENTARY MATERIAL

Rosmarinus officinalis L. Leaf Extracts and Their Metabolites Inhibit the Aryl Hydrocarbon Receptor (AhR) Activation In Vitro and in Human Keratinocytes: Potential Impact on Inflammatory Skin Diseases and Skin Cancer

Panagiotis Kallimanis [1], Ioanna Chinou [1,*], Angeliki Panagiotopoulou [2], Anatoly A. Soshilov [3], Guochun He [3], Michael S. Denison [3,†] and Prokopios Magiatis [1,3,*]

1 Laboratory of Pharmacognosy and Chemistry of Natural Products, Department of Pharmacy, National and Kapodistrian University of Athens, Panepistimiopolis Zografou, 15771 Athens, Greece; pgkallimanis@gmail.com

2 Institute of Biosciences and Applications, National Centre for Scientific Research "Demokritos", , Patr. Gregoriou E & 27 Neapoleos Str., Agia Paraskevi, 15310 Athens, Greece; apanagio@bio.demokritos.gr

3 Department of Environmental Toxicology, University of California, Davis, CA 95616, USA; anatoly.soshilov@oehha.ca.gov (A.A.S.); gchhe@ucdavis.edu (G.H.); msdenison@ucdavis.edu (M.S.D.)

* Correspondence: ichinou@pharm.uoa.gr (I.C.); magiatis@pharm.uoa.gr (P.M.); Tel.: +30-210-727-4052 (P.M.)

† The article is dedicated to the memory of Professor Michael S. Denison, who passed away during the reviewing procedure.

Preparation of extracts and isolation of 4′,7-*O*-dimethylapigenin (1), 7-*O*-methyl-*epi*-rosmanol (2), carnosol (3), carnosic acid (4) and betulinic acid (5) from *Rosmarinus officinalis* L. leaf

Standards and reagents

Methanol was of analytical grade purity and supplied by Sigma-Aldrich, CD_3OD and $CDCl_3$, Silica gel 0.040-0.063 mm, TLC (20×20 cm), normal and reverse phase, by Merck.

Preparation of the Rosemary dry Extracts

Three different dry extracts from rosemary leaves (ROS) were prepared using methanol in different time of extraction. The preparation method was the following: 100 g of the powdered dry leaf of rosemary was treated with the solvent, in a 1:10 ratio (100 g of dry leaf and 1L of solvent), for a specific time period (12 hours, 7 days and 2 months) in an opaque recipient. On the appropriate time the plant material was separated from the liquid part by filtration. Then, the volume of the solution was restored at the original volume and 2 L of distilled water were added. The formed precipitate and the supernatant were separated by filtration. The precipitate was dried and finally powdered to afford the corresponding rosemary dry extract, while the supernatant was collected and stored.

Processing of rosemary dry extracts

Maceration for 12 h in methanol

After 12 h of maceration of dry leaves (100 g) we obtained a dry extract **[ROS-12h]** (10.11 g) and the corresponded supernatant (3L).

Carnosol was isolated by pTLC normal phase, with 25.0 mg of **[ROS-12h]** and eluents CH_2Cl_2: CH_3OH= 97:3 v/v, affording carnosol (3 mg) as a white crystal, identified based on its NMR data.

1.29 g of [**ROS- 12h**] were subjected to column chromatography on silica gel (51 x 3 cm; silica gel 20.0 g) and eluted with cyclohexane (CHx)-ethyl acetate (EtOAc) as eluent with increasing ethyl acetate content (gradually from 100% of CHx to CHx: EtOAc = 50: 50% v/v) to provide 134 fractions (1-5, 40 mL; 6-134, 15 mL). Fractions 36-40 (47.6 mg) contained carnosic acid identified by NMR.

After 48 h of rest of the fractions 47-53 we observed the formation of colorless clear crystals. The crystals were then washed twice with 10 mL of mixture Chx: EtOAc = 80: 20 v/v. The crystals were then dissolved in methanol using the ultrasonic water bath. Then the solvent was evaporated in the rotary evaporator under reduced pressure, to provide 3.9 mg. The crystals were then spectroscopically analyzed by 1D ^{1}H-NMR and ^{13}C-NMR spectroscopy on $CDCl_3$ and CD_3OD. The crystals were identified as betulinic acid.

Maceration for 7 days

After 7 days of maceration of dry leaves we obtained a dry extract **[ROS-7d]** (9.83g) and the corresponded supernatant (3 L)

200mL of the supernatant were concentrated in vacuum till dryness (252.30 mg) and performed reverse phase preparative TLC with 25 mg and system of eluents CH_3OH: H_2O = 75: 25 v/v to yield 7-*O*-methyl-*epi*-rosmanol (3 mg)

Normal phase preparative TLC with 20.6 mg of **[ROS-7d]** and CH_2Cl_2: CH_3OH= 97:3 v/v as eluents afforded carnosol (2 mg)

1.20 g of [**ROS- 7d**] extract was subjected to column chromatography on silica gel (51 X 2,5 cm; silica gel 20 g) and eluted with CHx-EtOAc as eluent with increasing ethyl acetate content (gradually from 100% of CHx to CHx: EtOAc = 50: 50% v/v to provide 133 fractions (1-5, 40 mL; 6-125, 15 mL; 126-133, 50 mL). Fractions 51-60 (145.8mg) contained were combined subjected to pTLC on fluorescent silica gel plates using solvent system dichloromethane (CH_2Cl_2): methanol (CH_3OH) = 97:3 v/v giving two zones. One zone was scrapped off and eluted with ethyl acetate and the solvent was distilled off. Crystallization of the obtained residue from ethyl acetate yielded 4 mg of white crystals, recognized as carnosol.

After 48 hours of rest of the fraction 68-73 we observed formation of colorless clear crystalls as in the previous CC with the [**ROS- 12h**]. Following the same procedure as before we identified that the crystals were betulinic acid (11 mg).

Maceration for 2 months

Rosemary extract after 2 months of maceration **[ROS-2m]** (9.10 g)

pTLC normal phase, with 25 mg of **[ROS-2m]** and eluents CH_2Cl_2: CH_3OH= 97:3 v/v, led to the isolation of 7-methoxy-epirosmanol (2 mg) as a white crystal, identified based on its NMR data

1.2 g **[ROS-2m]** extract was applied to a silica gel column (3 X 51 cm; silica gel 20 g) and eluted using gradient of cyclohexane-ethyl acetate in order of increasing polarity, from 100% of CHx to CHx: EtOAc = 50: 50% v/v. 126 fractions (1-5, 40 mL; 6-21, 20mL; 22-126, 15 mL) were collected and combined according to their thin layer chromatography profiles resulting in 5 fractions, which were labeled I to V. 15.5 mg of fraction I (78.5 mg) were subjected to pTLC on fluorescent silica gel plates using CH_2Cl_2: CH_3OH = 97:3 v/v giving two zones who were scrapped off and eluted with ethyl acetate and the solvent was distilled off. Crystallization of the obtained residues from ethyl acetate yielded 4 mg of white crystals, recognized as 7-*O*-methyl-*epi*-rosmanol and 1 mg of 4′,7-*O*-dimethyl-apigenin as white crystals. After, 14.90 mg of fraction II (71.30 mg) were subjected to pTLC on fluorescent silica gel plates using solvent system CH_2Cl_2: CH_3OH = 97:3 v/v giving 6 zones who were scrapped off and eluted with ethyl acetate and the solvent was

distilled off. In zone A crystallization of the obtained residue of A zone from ethyl acetate yielded 4.90 mg of white crystals, recognized as 7-*O*-methyl-*epi*-rosmanol.

NMR spectroscopy

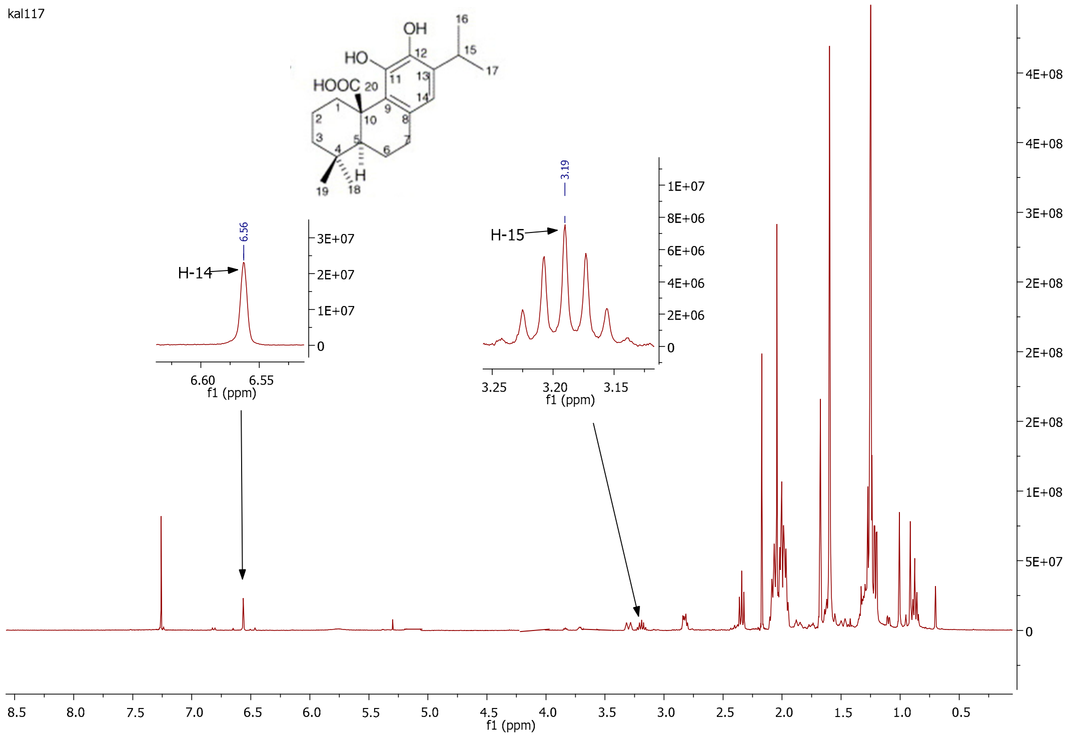

Figure S1. D ^{1}H-NMR spectra of CA in $CDCl_3$.

Table S1. ^{1}H-NMR, 13C-NMR of CA in $CDCl_3$.

Position	δ (ppm)	Integration	Multiplicity	J (Hz)	δc (ppm)
1	-				34.51
2	α 2.00<br>β 1.48	1<br>1	m<br>br d	 13.3	23.65
3	α 2.80-2.85<br>β 1.3-1.36	1<br>1	m<br>m		46.13
4	-	-	-	-	38.05
5	1.74	1	m		53.24
6	α 1.86<br>β 2.34	1<br>1	d<br>t	13.20<br>7.50	59.13
7	α 2.81<br>β 3.30	1<br>1	m<br>br d	 14.00	36.27
8	-	-	-	-	133.78
9	-	-	-	-	135.22
10	-	-	-	-	48.90
11	-OH 6.99	1	br s		148.5
12	-OH 5.76	1	br s		144.21
13	-	-	-	-	132.13
14	6.56	1	s		120.18
15	3.19	1	sept		29.14
16	1.22	3	d	2.9	25.21

17	1.20	3	d	3.2	26.32
18	1.01	3	s		34.87
19	0.91	3	s		23.43
20	-	-	-	-	184.19

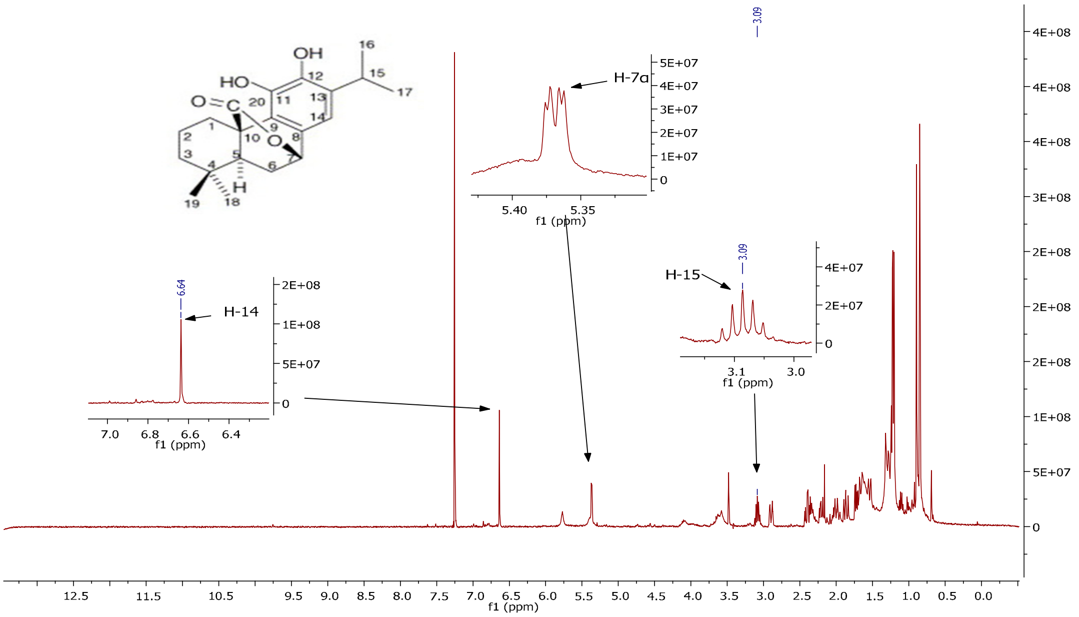

Figure S2A. 1D ^{1}H-NMR spectra of CS in $CDCl_3$.

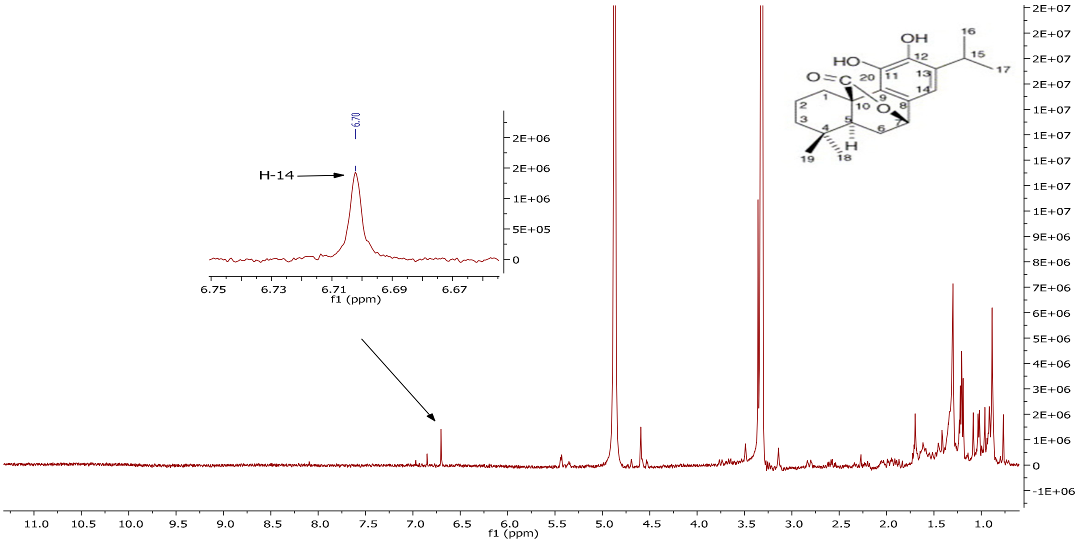

Figure S2B. 1D ^{1}H-NMR spectra of CS in CD_3OD.

Table S2A. ^{1}H-NMR, ^{13}C-NMR of CS in CDCl3.

Position	δ (ppm)	Integration	Multiplicity	J (Hz)	δC (ppm)
1	α 2.39	1	td	4.3, 13.3	29.51
1	β 2.90	1	brd	13	
2	α 1.65	1	m		19.32
2	β 2.0	1	dt		
3	α 1.29	1	dd	3.5, 13.2	41.20

3	β 1.54	1	brd	13.5	
4	-	-	-		34.74
5	1.72	1	q	7.0	45.65
6	α 2.20<br>β 1.87	1<br>1	m<br>td		29.93
7	α 5.37	1	dd	1.4, 4.0	78.33
8	-	-	-		132.38
9	-	-	-		121.44
10	-	-	-		48.16
11	-OH, 5.77	1	brs		141.78
12					141.52
13	-	-	-		132.93
14	6.64	1	s		112.34
15	3.09	1	sept	7	27.42
16	1.21	3	d	2.8	22.63
17	1.22	3	d	2.8	22.58
18	0.85	3	s		31.93
19	0.90	3	s		19.77
20	-	-	-		176.16

Table S2B. 1D 1H-NMR, 13C-NMR of CS in CD_3OD.

Position	δ (ppm)	Integration	Multiplicity	J (Hz)	δ_C (ppm)
1	α 2.58<br>β 2.81	1<br>1	ddd<br>brd	4.2, 14.0<br>13.9	30.36
2	α 1.63<br>β 1.92	1<br>1	m<br>m		20.07
3 	 β 1.53	 1	 brd	 14.0	42.59
4	-	-	-		35.74
5		1			47.36
6<br>6	α 1.86<br>β 2.19	1<br>1	m<br>ddd	 4.0, 5.8, 14.0	30.94
7	α 5.44	1	dd	1.7, 3.7	78.47
8	-	-	-		129.31
9	-	-	-		123.03
10	-	-	-		49.87
11	-	-	-		139.95
12	-	-	-		144.97
13	-	-	-		136.31
14	6.70	1	s		112.84
15	3.24	1	m		28.62
16	1.20	3	d	7.0	23.20
17	1.23	3	d	4.5	23.23
18	0.88	3	s		32.94
19	0.87	3	s		20.16
20	-	-	-		179.81

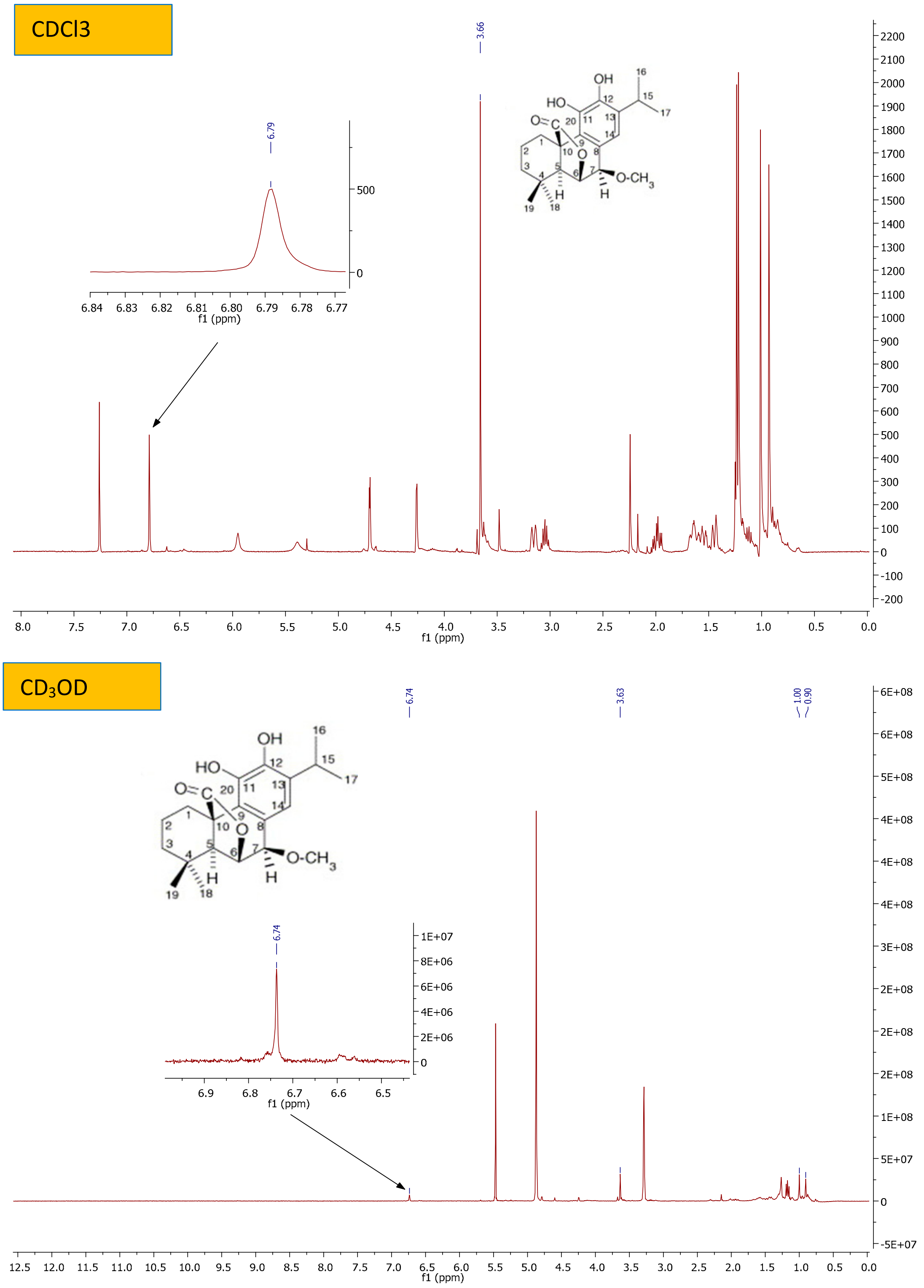

Figure S3. Spectra ^{1}H-NMR of 7MER in $CDCl_3$ and in CD_3OD.

Table S3A. ^{1}H-NMR, ^{13}C-NMR of 7MER in $CDCl_3$.

Position	δ (ppm)	Integration	Multiplicity	J (Hz)	δC (ppm)
1	α, 1.99	1	td	5.0, 13.5	27.04
	β, 3.16	1	brd	13.80	
2	α, 1.64	1	m		19.76
	β, 1.56	1	m		

3	α, 1.45<br>β, 1.12	1<br>1	brd<br>m	13.0	38.08
4	-	-	-	-	31.35
5	2.24	1	s		51.14
6	4.71	1	d	3.0	75.03
7	4.26	1	d	3.0	77.61
8	-	-	-	-	125.93
9	-	-	-	-	124.85
10	-	-	-	-	47.26
11	-OH, 5.40	1	br s		143.54
12	-OH, 5.95	1	br s		142.09
13	-	-	-	-	135.67
14	6.79	1	s		120.8 5
15	3.05	1	sept	7.20	27.13
16	1.23	3	d	7.50	22.25
17	1.23	3	d	7.50	22.52
18	0.93	3	s		22.07
19	1.01	3	s		31.5
20	-	-	-	-	179.93
-OCH3	3.66	3	s		58.27

Table S3B. ^{1}H-NMR, ^{13}C-NMR of 7MER in CD_3OD.

Position	δ (ppm)	Integration	Multiplicity	J (Hz)	$δ_C$ (ppm)
1	α, 1.94<br>β, 3.29	1<br>1	td<br>m	5.0, 13.70	28.32
2	α, 1.48<br>β, 1.57	1<br>1	m<br>m		19.93
3	α, 1.43	1	brd	13.00	38.91
4	-	-	-	-	32.96
5	2.15	1	s		56.54
6	4.79	1	d	3.20	80.13
7	4.25	1	d	3.20	75.78
8	-	-	-	-	128.64
9	-	-	-	-	125.55
10	-	-	-	-	47.87
11	-	-	-	-	145.83
12	-	-	-	-	142.71
13	-	-	-	-	138.31
14	6.73	1	s		120.27
15	3.19	1	sept	7.20	28.45
16	1.18	3	d	7.00	23.82
17	1.16	3	d	7.00	31.35
18	0.90	3	s		32.27
19	1.00	3	s		22.54
20	-	-	-	-	20.91
-OCH3	3.63	3	s		56.37

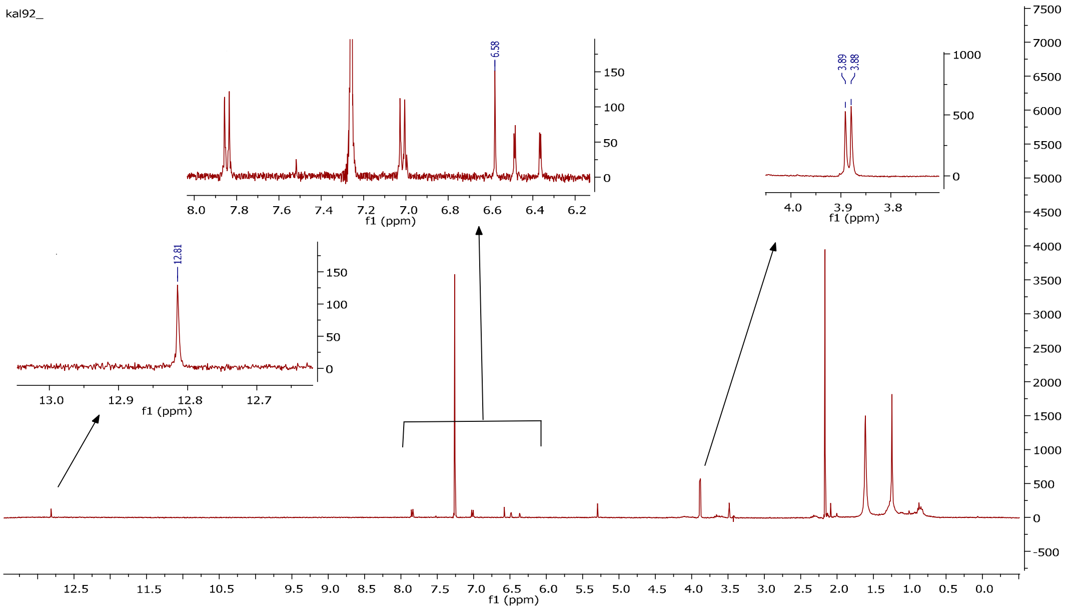

Figure S4. ^{1}H-NMR spectra of DMA in $CDCl_3$.

Table S4. ^{1}H-NMR, ^{13}C-NMR of DMA in $CDCl_3$.

Position	δ (ppm)	Integration	Multiplicity	J (Hz)	δ_C (ppm)
2	-	-	-	-	162.9
3	6.58	1	s	-	104.8
4	-	-	-	-	182.7
5, -OH	12.81	1	brs	-	158.1
6	6.49	1	d	2.5	98.4
7	-	-	-	-	165.8
8	6.37	1	d	2.5	93.1
9	-	-	-	-	162.5
10	-	-	-	-	105.8
7, $-OCH_3$	3.89	3	s	-	56.2
1′	-	-	-	-	123.9
2′	7.85	1	d	9	128.2
3′	7.02	1	d	9	113.9
4′	-	-	-	-	164.1
5′	7.02	1	d	9	113.9
6′	7.85	1	d	9	128.2
4′, $-OCH_3$	3.88	3	s	-	55.53

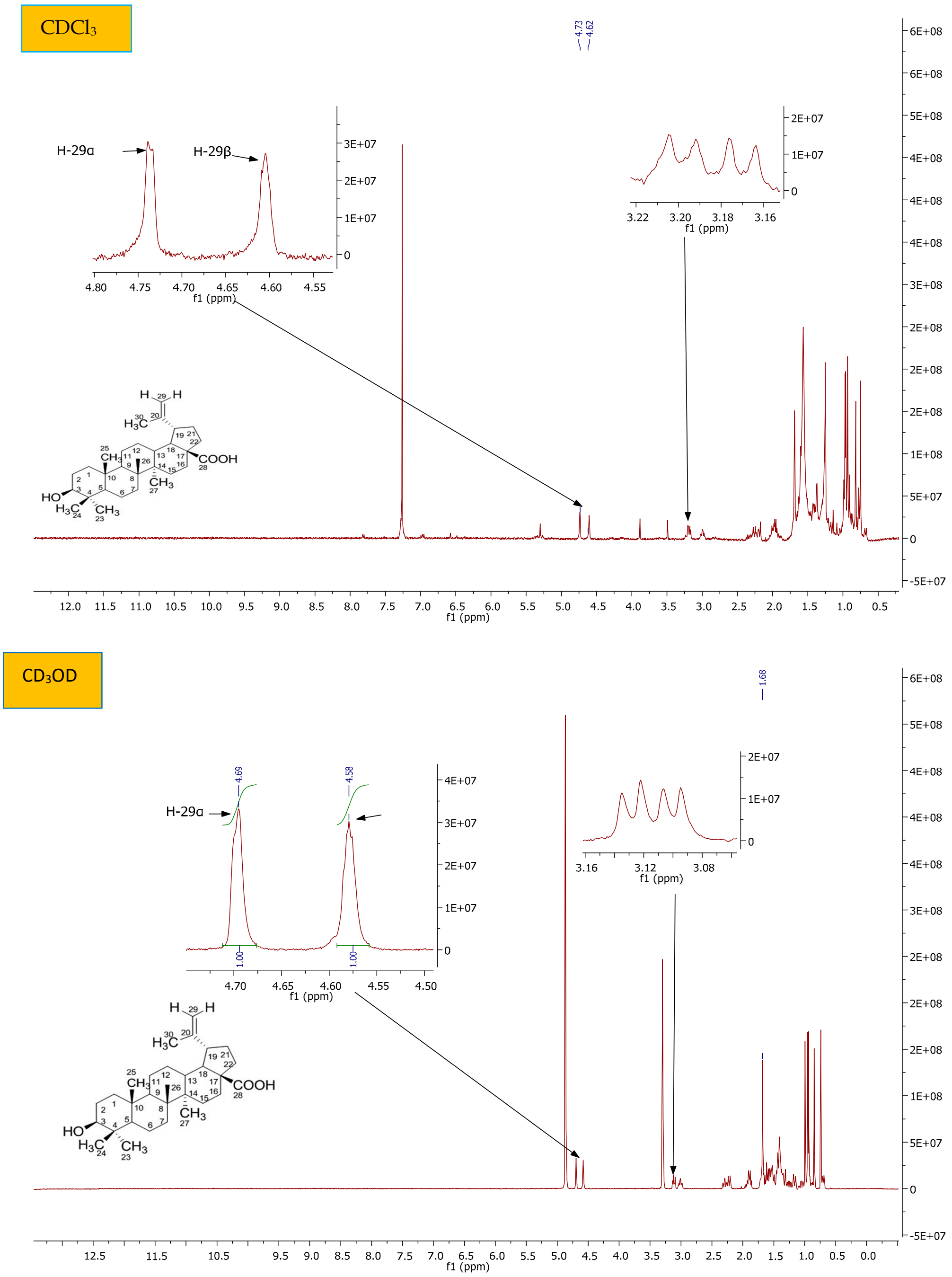

Figure S5 ^{1}H-NMR spectra of betulinic acid.

Table S5A. ^{1}H-NMR, ^{13}C-NMR of BA in $CDCl_3$.

Position	δ (ppm)	Integration	Multiplicity	J (Hz)	δ_C (ppm)
1					38.69
2					27.47
3	3.18	1	dd	5.20, 12.0	78.79
4	-	-	-	-	38.74
5	0.68	1	brd		55.30
6	1.40	2	m		18.23

7					34.27
8	-	-	-	-	40.61
9	1.25	1	br s		50.39
10	-	-	-	-	37.18
11	1.25	2	br s		20.83
12	α 1.69	1	s		25.48
13	2.20	1	m		38.31
14	-	-	-	-	42.44
15					30.52
16					32.15
17	-	-	-	-	56.22
18					47.15
19	2.99	1	m		46.57
20	-	-	-	-	150.51
21	α 1.42	1	m		29.66
22	1.97	2	m		37.06
23	0.96	3	s		27.90
24	0.75	3	s		15.29
25	0.82	3	s		15.95
26	0.93	3	s		16.01
27	0.97	3	s		14.62
28					179.40
29	α 4.74;<br>β 4.61	1;<br>1	br s;<br>br s		109.56
30	1.69	3	br s		19.27

Table S5B. 1D ^{1}H-NMR, ^{13}C-NMR of BA in CD_3OD.

Position	δ (ppm)	Integration	Multiplicity	J (Hz)	δ_C (ppm)
1	α 0.88	1	m		38.75
2	β 1.55	1	m		27.69
3	3.11	1	dd	5.20, 12.0	77.99
4	-	-	-	-	39.07
5	0.70	1	br d	8.85	55.41
6					18.46
7					34.49
8	-	-	-	-	40.71
9	1.36	1	m		50.48
10	-	-	-	-	37.23
11					20.92

12	α 1.04<br>β 1.88	1<br>1	dd<br>m	4.75<br>13.0	25.62
13	2.29	1	td	4.0 ,12.0	38.15
14	-	-	-	-	42.47
15	α 1.25	1	m		30.62
16	α 1.55<br>β 2.22	1<br>1	m<br>m		32.23
17	-	-	-	-	55.96
18	1.61	1	t	11.30	49.09
19	3.01	1	m		47.13
20	-	-	-	-	150.81
21	α 1.41	1	m		29.74
22					36.80
23	0.96	3	s		28.31
24	0.74	3	s		15.91
25	0.85	3	s		16.33
26	0.99	3	s		16.41
27	0.94	3	s		14.93
28					177.87
29	α 4.69<br>β 4.58	1<br>1	s<br>s		110.13
30	1.68	3	s		19.41

Table S6. Concentration of major metabolites in *Rosmarinus officinalis* extracts measured by qNMR.

R. Offici-	**Sol-vent**	**Mac-era-**	**Compounds (mg±SD / g extract)**				
			**1**	**2**	**3**	**4**	**5**
**R1**	**Ethanol 96º**	48 h	-	-	23.5±3.1	46.8±5.3	124.6±13.4
**R2**	Ethanol 96º	14 d	-	-	38.0±4.0	31.9±3.1	157.7±17.1
**R3**	Methanol	48 h	1.0±0.1	-	44.4±5.2	69.4±7.2	111.8±13.5
**R4**	Methanol	7 d	1.7±0.2	2.2±0.3	50.5±4.3	42.1±6.1	106.3±10.4
**R5**	Isopropa-nol	14 d	-	-	12.7±2.5	20.9±3.1	69.5±5.1

1: DMA , 2:7MER, 3: CS, 4: CA, 5: BA.

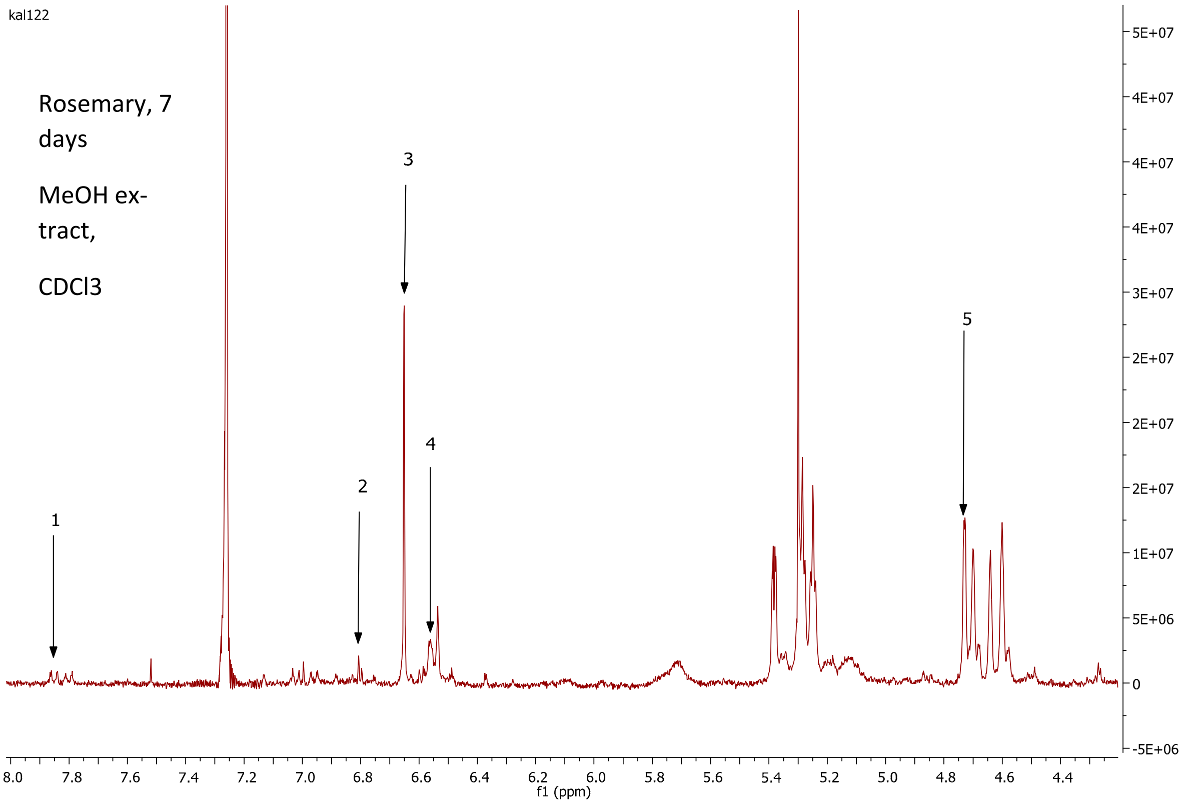
kal122
Rosemary, 7 days
MeOH extract,
CDCl3
1
2
3
4
5
f1 (ppm)

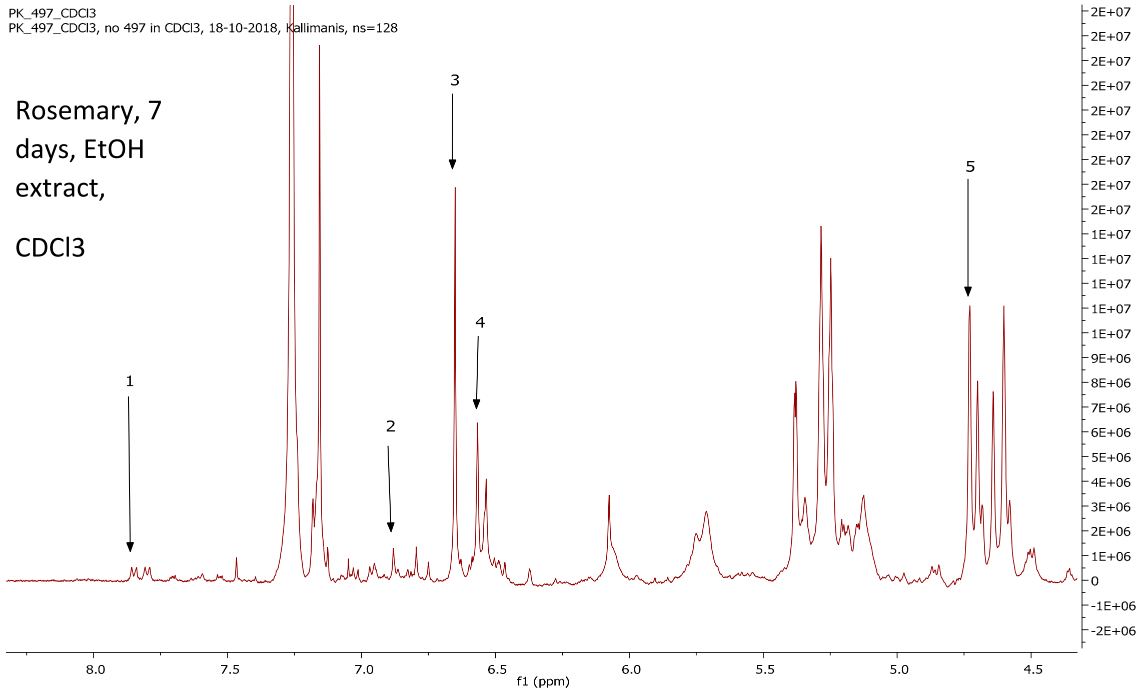
PK_497_CDCl3
PK_497_CDCl3, no 497 in CDCl3, 18-10-2018, Kallimanis, ns=128
Rosemary, 7 days, EtOH extract,
CDCl3
1
2
3
4
5
f1 (ppm)

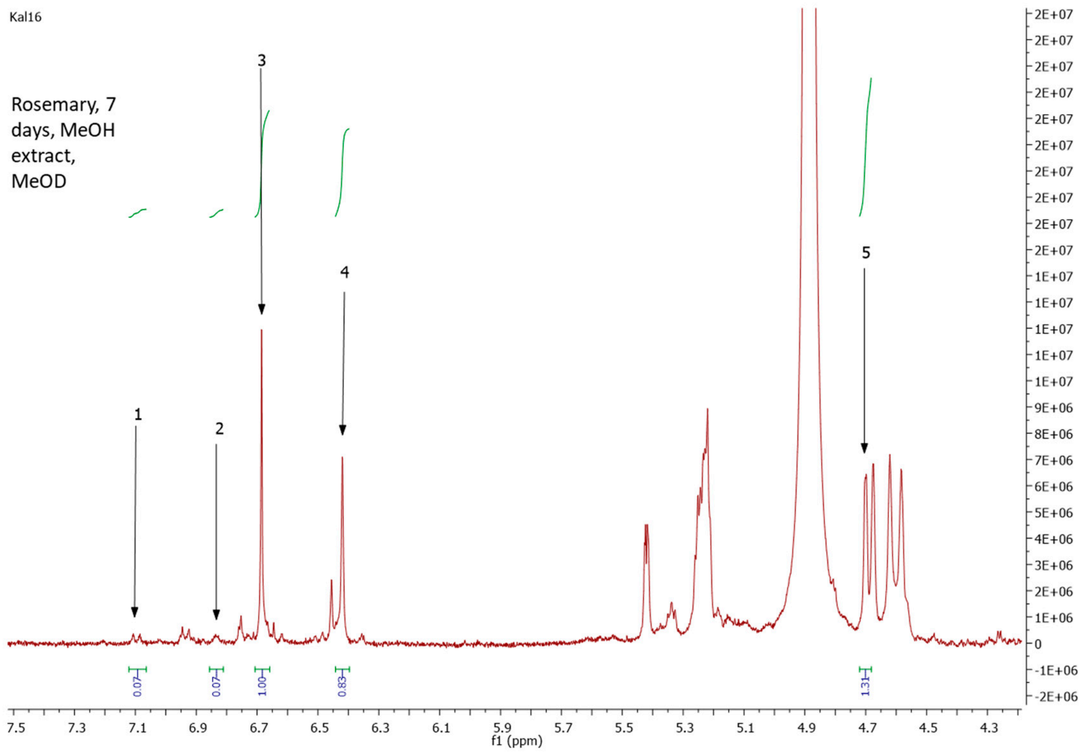

Figure S6. NMR spectra of ROEs showing the peaks used for quantitation for each metabolite.
